# Transformation of novel TiOF_2_ nanoparticles to cluster TiO_2_-{001/101} and its degradation of tetracycline hydrochloride under simulated sunlight

**DOI:** 10.1039/d0ra08476j

**Published:** 2020-11-24

**Authors:** Yue Jian, Huayang Liu, Jiaming Zhu, Yaqiong Zeng, Zuohua Liu, Chentao Hou, Shihua Pu

**Affiliations:** Chongqing Academy of Animal Sciences Chongqing 402460 China pu88962@126.com; College of Geology and Environment, Xi'an University of Science and Technology Xi'an 710054 China 807484470@qq.com; Scientific Observation and Experiment Station of Livestock Equipment Engineering in Southwest, Ministry of Agriculture and Rural Affairs Chongqing 402460 China

## Abstract

The anatase type cluster TiO_2_-{001/101} was rapidly generated by a one-step hydrothermal method. The transformation process of coral-like TiOF_2_ nanoparticles to cluster TiO_2_-{001/101} was investigated for the first time, and the sensitization between cluster TiO_2_-{001/101} and tetracycline hydrochloride (TCH) was also discussed. The degradation rate of TCH by cluster TiO_2_-{001/101} under simulated sunlight was 92.3%, and the total removal rate was 1.76 times that of P25. Besides, cluster TiO_2_-{001/101} settles more easily than P25 in deionized water. The study showed that cluster TiO_2_-{001/101} derived from coral-like TiOF_2_ nanoparticles had a strong adsorption effect on TCH, which was attributed to the oxygen vacancy (O_v_) and {001} facets of cluster TiO_2_-{001/101}. The strong adsorption effect promoted the sensitization between cluster TiO_2_-{001/101} and TCH, and widened the visible light absorption range of cluster TiO_2_-{001/101}. In addition, the fluorescence emission spectrum showed that cluster TiO_2_-{001/101} had a lower luminous intensity, which was attributed to the heterojunction formed by {001} facets and {101} facets that reduces the recombination rate of carriers. It should be noted that cluster TiO_2_-{001/101} still has good degradation performance for TCH after five cycles of degradation. This study provides a new idea for the synthesis of cluster TiO_2_-{001/101} with high photocatalytic performance for the treatment of TCH wastewater.

## Abstract 2

Degradation of tetracycline hydrochloride by cluster TiO_2_-{001/101} under simulated sunlight.

## Graphical abstract

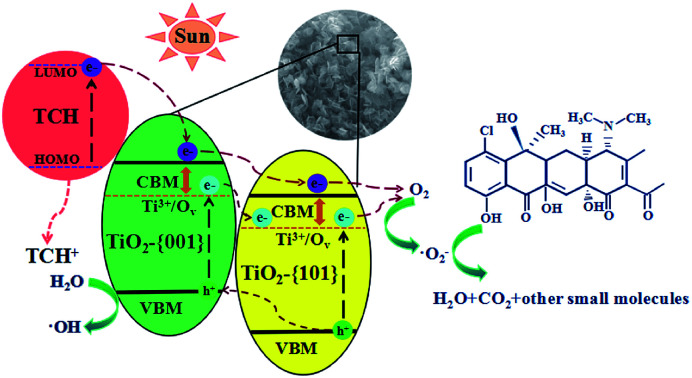

## 1. Introduction

Antibiotics are widely used around the world. TCH is a typical broad-spectrum tetracycline antibiotic, which is produced by actinomycetes and widely used as a growth promoter in the treatment of human diseases and animal feeding.^1^ However, because the naphthol ring in the TCH structure can't be completely metabolized by humans and animals, a large amount of TCH absorbed is excreted into various water bodies through feces and urine.^2^ Drinking water containing TCH for a long time will cause a series of diseases such as bacterial disorders, gastrointestinal reactions and mycoplasma pneumonia, and affect the growth of teeth and bones.^3–5^

In recent years, some methods have been used to treat TCH wastewater, such as microbial degradation,^6,7^ adsorption,^8,9^ electrochemical method,^10^ membrane separation method,^11,12^ photocatalytic degradation.^13–16^ Among these methods, photocatalytic water purification technology is considered to be the most promising water treatment technology due to its economic, efficient and clean environment.^17–20^

TiO_2_ is widely used in environmental protection,^18^ biomedicine,^21^ solar cells^22^ and many other fields due to its advantages of simple control, low cost and non-toxic.^23^ Especially in the photocatalytic treatment of pollutants showed excellent performance. However, it has a wide energy band gap (3.0–3.2 eV, which means it only reacts to UV) and a high carrier recombination rate, which hinders its application in sunlight.^24,25^ So far, many efforts have been made to make up for these shortcomings, such as metal doping,^26–28^ non-metal doping,^29–31^ dye sensitization,^32–34^ and building heterogeneous semiconductor composite.^35–38^ Among these methods, the construction of semiconductor heterojunction seems to be an effective method to improve charge separation. However, the high-quality tight interface requirements required by heterojunction make the construction more difficult.^39^ Although dye sensitization can improve the response of TiO_2_ to visible light and enhance the photocatalytic activity. However, due to the weak binding force between organic dyes and TiO_2_, if the dyes can not timely supplement the lost electrons in the system, the dyes will fall off from the TiO_2_ and form competitive degradation with the target pollutants, which will reduce the degradation efficiency of the target pollutants.^40^ Doping is the most commonly used method to enhance the photocatalytic activity of TiO_2_ because of its simple method.^41–44^

It is worth noting that fluorine (F) is a special anionic dopant. The existence of fluorine forms –Ti–F–Ti– and Ti^3+^ species to replace lattice oxygen to form O_vs_.^45^ The formation of Ti^3+^/O_vs_ is an effective and environmentally friendly strategy to improve visible light absorption and pollutant adsorption. Moreover, the existence of O_v_ can form an electron trap, which greatly reduces the composite rate of electrons and holes, thus effectively enhancing the photocatalytic activity of TiO_2_.^46,47^ Furthermore, F, as an end-capping agent, promotes the growth of TiO_2_-{001} facet.^48^ For instance, Han *et al.*^49^ using tetrabutyl titanate and hydrofluoric acid as precursors, the F–TiO_2_ nanosheets with 89% TiO_2_-{001} facets were obtained by hydrothermal treatment. Its photocatalytic activity is much higher than that of Degussa P25. Up to now, extensive experimental and theoretical studies have shown that the {001} facet with high surface energy has higher catalytic activity than the {101} facet with thermodynamic stability.^50,51^ This is because a low coordination number of exposed atoms and a high density of unsaturated Ti atoms on the {001} facet are in favor of the dissociation and adsorption of reactants.^52^ Lu *et al.*^53^ demonstrated that the {001} facets of anatase TiO_2_ nanocrystals exhibited much better photocatalytic activity than that of {101} facets of anatase TiO_2_ nanocrystals toward photocatalytic oxidation of water and organic compounds with different functional groups (*e.g.*, –OH, –CHO, –COOH). Besides, Chen *et al.*^54^ found that anatase TiO_2_ microcrystals with highly exposed {001} facets have strong photocatalytic decomposition ability for gaseous styrene. Although the surface has very high surface energy, maximizing the percentage of exposed {001} area might not be a good direction. Khalil *et al.*^55^ demonstrated that an appropriate proportion of exposed {001} and {101} facet, which forms a “surface heterojunction”. In the photocatalytic reactions, photogenerated electrons tend to transfer to the {101} facet with lower energy, and thus accumulating on the {101} facet, while photogenerated holes tend to accumulate on the {001} facet with high energy. This feature can effectively promote the separation of photogenerated electron–hole pairs, thereby improving their photocatalytic performance.^56^ Therefore, it is necessary to explore how to synthesize TiO_2_ with {001} and {101} facet.

In many methods of synthesing TiO_2_, solvothermal reaction conditions are relatively mild, and it can control the morphology of materials well.^22,28,57^ In the typical synthesis methods, tetrabutyl titanate was used as titanium source, ethanol as an inhibitor and hydrofluoric acid as end-capping agent. After hydrothermal treatment, TiO_2_ nanosheets with {001} and {101} facets co-exposed could be synthesized. Interestingly, TiOF_2_ is sometimes formed during the formation of TiO_2_-{001}. In the previously reported X-ray diffraction characterization, it can be seen that TiOF_2_ has two crystal forms, corresponding to no. 08-0060 and no. 01-0490 in JCPDS database.^25,58^ The TiOF_2_ corresponding to JCPDS no. 08-0060 shows regular cubic morphology, while the TiOF_2_ corresponding to JCPDS no. 01-0490 shows irregular morphology of nanoparticles. Over the past ten years, some researchers have carried out extensive research on cubic TiOF_2_. For instance, Zhang *et al.*^59^ synthesized 3D hollow nanospheres with {001} main exposure facet using cubic TiOF_2_ as a template, which improved the response of TiO_2_ to visible light. Zhang *et al.*^58,60,61^ controlled the formation process of TiO_2_-{001} nanosheets under different hydrofluoric acid dosage, reaction time and reaction temperature. It is found that cubic TiOF_2_ is an important intermediate in the hydrothermal synthesis of TiO_2_-{001} nanosheets. Shi *et al.*^62^ studied the transformation process of cubic TiOF_2_ to TiO_2_-{001/101} at different calcination temperatures and proposed the possible transformation mechanism of TiOF_2_ to TiO_2_-{001/101}. However, anatase TiO_2_ nanosheets derived from cubic TiOF_2_ are relatively large and easy to aggregate, which undoubtedly reduces the exposure area of TiO_2_-{001} and leads to a low density of active sites, which inevitably increases the possibility of carrier recombination. Therefore, to further improve the photocatalytic activity of anatase TiO_2_, it is necessary to synthesize small-sized TiO_2_ with {001} and {101} facet co-exposed. Compared with cubic TiOF_2_, few researchers pay attention to the TiOF_2_ nanoparticles corresponding to JCPDS no. 01-0490. In our previous research,^25^ this crystal type of TiOF_2_ nanoparticles were successfully synthesized, and found that it can also transform into anatase TiO_2_-{001} in hydrothermal reaction, and the obtained TiO_2_/TiOF_2_ hybrid has close interface contact and layered structure. Compared with cubic TiOF_2_, this kind of TiOF_2_ nanoparticles has a large number of mesoporous structures and smaller size. To enrich the research system of TiOF_2_, it is necessary to explore the transition process of novel TiOF_2_ in hydrothermal reaction. Moreover, this kind of TiOF_2_ will be expected to transform into smaller TiO_2_-(001/101) nanosheets under certain conditions.

In this study, the transition process of novel TiOF_2_ nanoparticles to cluster TiO_2_-{001/101} in the hydrothermal reaction was discussed for the first time. Under the irradiation of simulated sunlight, the degradation rate of TCH by TiO_2_-{001/101} was 92.3%. Besides, the sedimentation performance of TiO_2_-{001/101} was investigated and compared with that of Degussa P25. Furthermore, the sensitization between TCH and TiO_2_-{001/101} was proposed for the first time, and the possible degradation pathway of TCH was discussed. This study provides an idea for the treatment of TCH wastewater.

## 2. Experimental

### 2.1. Chemicals

Tetrabutyl titanate (TBOT, A. R.), anhydrous ethanol (C_2_H_5_OH, A. R.), hydrofluoric acid (HF, A. R.) was purchased from Fuchen Chemical Reagent Factory, Tianjin, China. Degussa P25 (P25) was purchased from Beijing JiaHeng Technology Co., Ltd. Tetracycline hydrochloride (TCH) was provided by Aladdin Industrial Corporation (Shanghai, China), benzoquinone (BQ), methanol (MT), *tert*-butyl alcohol (*t*-BuOH) were purchased from Fuchen Chemical Reagent Factory, Tianjin, China. All reagents were used without further purification.

### 2.2. Synthesis of the photocatalysts

The experimental water is ultra-pure water. TiO_2_-{001/101} were synthesized by a one-step hydrothermal method. Add 30 mL anhydrous ethanol and 8 mL hydrofluoric acid into 100 mL polytetrafluoroethylene liner (Shanghai Qiuzuo Scientific Instrument Co., Ltd), stir for 10 min at 25 °C, and record the mixture as solution A. Take 30 mL tetrabutyl titanate, add it to solution A under stirring at the rate of 2 drops per second to form a white suspension, and stir the mixture at 25 °C for 2 h. Then, the polytetrafluoroethylene was placed in a high-pressure reactor (Shanghai Qiuzuo Scientific Instrument Co., Ltd) and reacted at 150 °C for 4 h. After the system is naturally cooled to room temperature, the solid products are collected by centrifugation and washed with ethanol and ultra-pure water three times. The samples were dried at 60 °C and named T-4h. The above steps remained unchanged and the reaction time varied between 0.5 h, 1 h and 3 h to study the transition process of novel TiOF_2_ to TiO_2_-{001/101}. These products were recorded as T-0.5h, T-1h, T-3h. Besides, we put the photocatalyst (30 mg) into 100 mL TCH (10 mg L^−1^), stirred for 60 min under dark conditions, and then centrifuged and dried at 60 °C. The obtained samples were recorded as T-0.5h/TCH, T-1h/TCH, T-3h/TCH, T-4h/TCH and P25/TCH.

### 2.3. Characterization of the samples

The crystal structure of the samples was observed using X-ray diffraction (XRD; Bruker D8 Advance X-ray diffractometer) at 36 kV, 20 mA equipped with a Cu anode X-ray tube (Cu Kα X-rays, *λ* = 1.54056 Å). SEM and EDS (JSM7500F, Japan) were used to record the surface morphology and element distribution of the samples. Transmission electron microscopy (TEM) images were achieved with a JEOL JEM-2100 high-resolution transmission electron microscope. The specific surface area (*S*_BET_) and pore size analysis were performed by Brunauer–Emmett–Teller (BET) and Barrett–Joyner–Halenda (BJH) methods through a micromeritics JW-BK122W. The absorption characteristics of the samples were determined by UV-visible diffuse absorption spectroscopy (UV-Vis DRS, Shimadzu UV-2600, Japan). Fourier transform infrared spectrum (FT-IR) measurements, recorded in the range of 4000–400 cm^−1^, were performed in KBr pellet by Nicolet IS5 Spectrometer, USA. The photoluminescence spectrum of the photocatalyst was measured by a fluorescence spectrometer (Shimadzu-RF-6000, Japan) with the excitation wavelength was 300 nm. The chemical valence states of the samples were analyzed by X-ray photoelectron spectroscopy (XPS, ThermoFisher K-Alpha). Electron paramagnetic resonance (EPR) was implemented by the BrukerA 300 spectrometer operating at room temperature.

### 2.4. Photocatalytic activity measurement

The photocatalytic activity of the catalyst was tested by the degradation of TCH. First, at the maximum absorption wavelength of 357 nm, the initial absorbance of TCH solution (10 mg L^−1^) was measured by a UV-Vis spectrophotometer (Hitachi, UV-3900, Japan). After that, take 100 mL of TCH solution (10 mg L^−1^) in a measuring cylinder and put it into a 150 mL photocatalytic reaction tube (Ghx-1, Shanghai Qiaofeng Industrial Co., Ltd), weigh 30 mg of photocatalyst and add it into the TCH solution. The photocatalytic reaction tube was placed in the photocatalytic reactor (Ghx-1, Shanghai Qiaofeng Industrial Co., Ltd), and a 500 W xenon lamp (GXZ-500, Shanghai Jiguang Special Lighting Appliance actory) was used to simulate the degradation of TCH by sunlight. Before the xenon lamp is turned on, the solution is magnetically stirred in the dark for 60 min to ensure the adsorption balance, then turn on the xenon lamp to start the photodegradation test, take out 7 mL suspension every 10 min, and centrifugate at high speed to remove the catalyst, then analyze the remaining TCH concentration with UV-Vis spectrophotometer at the maximum absorption wavelength of 357 nm. After each photodegradation experiment, the sample was separated by a high-speed centrifuge (Tgl-16c, Shanghai Anting Scientific Instrument Factory), washed with distilled water, and ultimately dried at 60 °C for the next test. The initial concentration of TCH was changed from 10 mg L^−1^ to 20 mg L^−1^, 30 mg L^−1^ and 40 mg L^−1^ to explore the effect of different TCH concentrations on photocatalytic degradation. The dosage of the photocatalyst was changed from 30 mg to 10 mg, 20 mg and 40 mg to explore the effect of photocatalyst dosage on the degradation of TCH. Different scavengers (BQ, MT and *t*-BuOH) were used to trap the active components (·O^2−^, h^+^, ·OH) in the photocatalytic process. This test is similar to the photocatalytic degradation test, except that a certain amount of scavenger was added to the TCH solution, and then photocatalyst was added.

### 2.5. Sedimentation experiment

The settling behavior of T-4h and P25 in deionized water were studied in glass cylinders (100 mL, diameter 2.8 cm, height 24 cm Fuchen Chemical Reagent Factory, Tianjin, China). T-4h suspension (1 g L^−1^) was prepared with deionized water. The obtained T-4h suspension was homogenized by violent shaking and then poured into the cylinder. The settlement behavior of T-4h was observed by standing for 2 h, 10 h, 24 h. The settlement behavior of P25 was studied under the same conditions.

## 3. Results and discussion

### 3.1. Crystal structure analysis

**Fig. 1:**
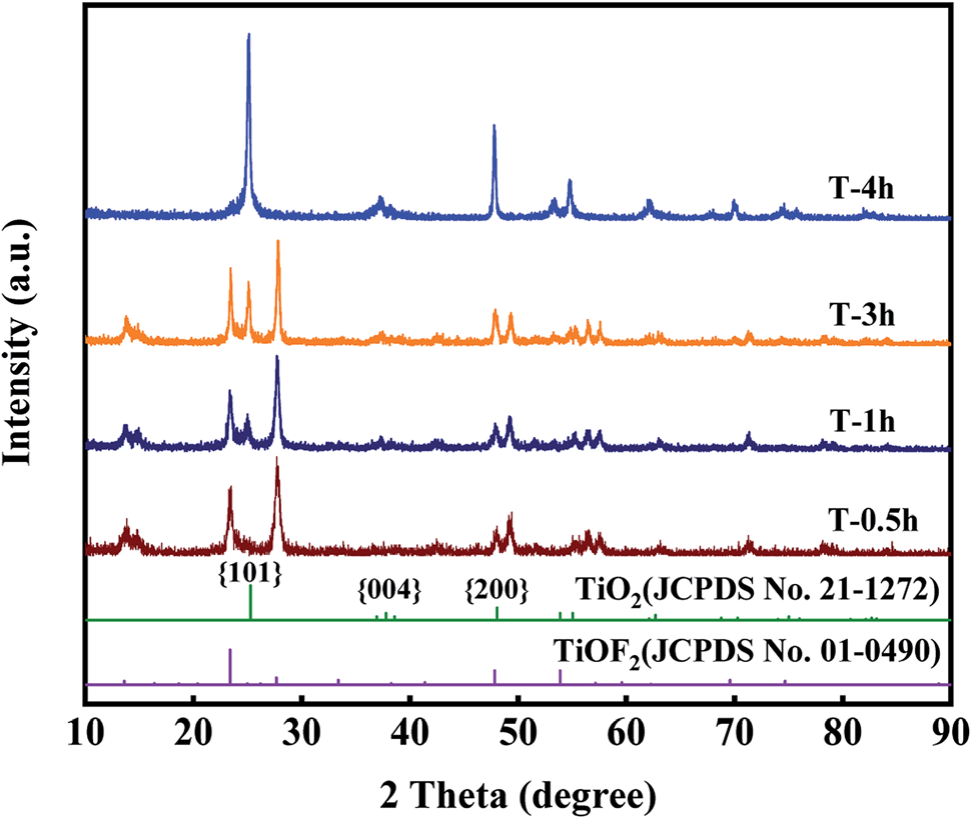
XRD patterns of the prepared samples.

Fig. 1 clearly shows the crystal structure of the prepared samples. For T-0.5h, the diffraction peaks at 2*θ* = 13.61°, 23.39°, 27.68°, 47.8°, 53.88°, 59.6°, 69.6°, 74.7° match with TiOF_2_ (JCPDS no. 01-0490), indicating that the novel TiOF_2_ was successfully prepared.^25^

For T-4h, the diffraction peaks at 2*θ* = 25°, 37.76°, 47.8°, 54.86°, 56.6°, 70.3°, 75°, 82.22° are attributed to the {101}, {004}, {200}, {105}, {211}, {220}, {215}, {224} planes of anatase TiO_2_ (JCPDS no. 21-1272), respectively.^30,32,38^ It can be seen that with the reaction time from 0.5 h to 4 h, the diffraction peak intensity of TiO_2_ {101} facet increases gradually, while the diffraction peak of TiOF_2_ completely disappears at 4 h, which indicates that TiOF_2_ has completely evolved into anatase TiO_2_. It should be noted that there is an obvious diffraction peak of {004} facet in T-4h, which is often referred to as the existence of {001} facet.^25,39^

In summary, the chemical reactions of the formation of TiO_2_-{001/101} nanosheets can be proposed. The first step is the hydrolysis of TBOT. The reaction of TBOT to form Ti(OH)_4_ is shown in eqn (1).^54^ Secondly, Ti(OH)_4_ can react with HF to produce TiOF_2_eqn (2).^25^ Finally, with the hydrothermal reaction, TiOF_2_ was hydrolyzed to anatase TiO_2_-{001/101}, as shown in eqn (3).^47^

1 Ti(OC_4_H_9_)_4_ + 4H_2_O → Ti(OH)_4_ + 4C_4_H_9_OH

2 Ti(OH)_4_ + 2HF → TiOF_2_↓ + 3H_2_O

3 TiOF_2_ + H_2_O → TiO_2_↓ + 2HF

### 3.2. Morphology analysis of photocatalyst

**Fig. 2:**
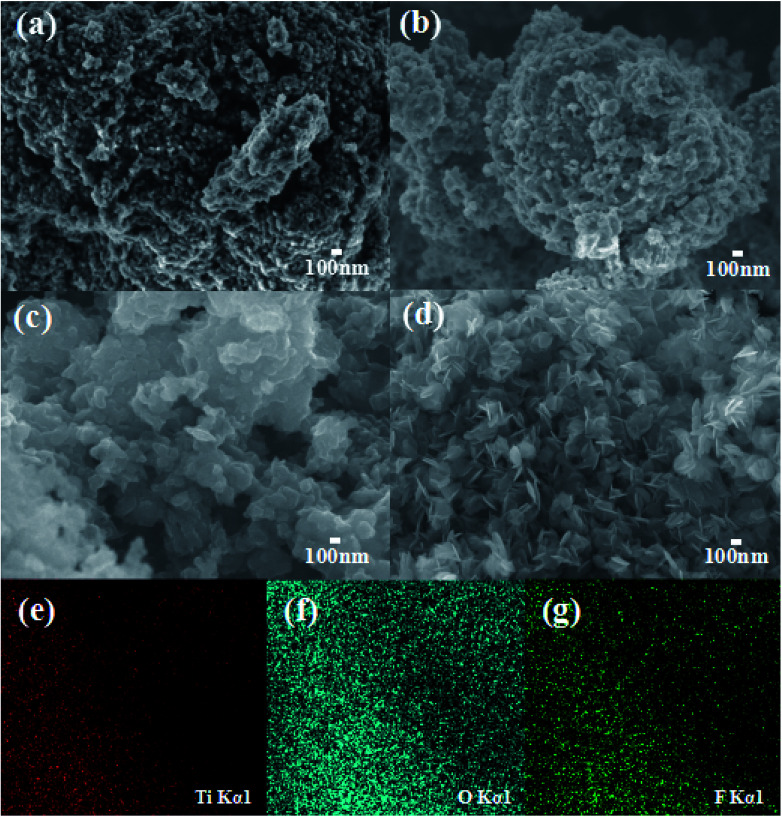
The high-magnification FE-SEM images of (a) T-0.5h, (b) T-1h, (c) T-3h, (d) T-4h; the element mapping of (e) Ti, (f) O, (g) F in T-4h.

Field emission scanning electron microscopy (FE-SEM) images of T-0.5h, T-1h, T-3h, T-4h are shown in Fig. 2. Fig. 2a shows the T-0.5h image. The novel of TiOF_2_ shows coral-like structure formed by the accumulation of spherical TiOF_2_ nanoparticles, which is completely different from the cubic TiOF_2_ that researchers have focused on before.^58–61^ The novel TiOF_2_ has many macroporous structures, which is consistent with the pore size analysis. With the extension of reaction time, the spherical TiOF_2_ nanoparticles tend to be flat, which may be due to the conversion of some TiOF_2_ to TiO_2_ (Fig. 2b). With the further hydrothermal reaction, it can be seen that the TiO_2_ nanosheets derived from the surface of TiOF_2_ are closely interlaced (Fig. 2c). By XRD analysis, most of the {101} and a few {001} facets of TiO_2_ have been co-exposed, and a close interface contact is formed between the TiO_2_-{001/101} and the untransformed TiOF_2_. When the reaction was further extended to 4 h, only TiO_2_-{001/101} nanosheets were formed (Fig. 2d). TiO_2_ nanosheets with an average diameter of about 200 nm are interlaced with each other to form clusters, which is conducive to the migration and transformation of electrons and holes between {001} and {101} facets. To further describe the element distribution of T-4h, elemental mapping analysis (Fig. 2e–g) of T-4h was performed. It is proved the existence of Ti, O, F elements and the mapping image matches well with the SEM image.

**Fig. 3:**
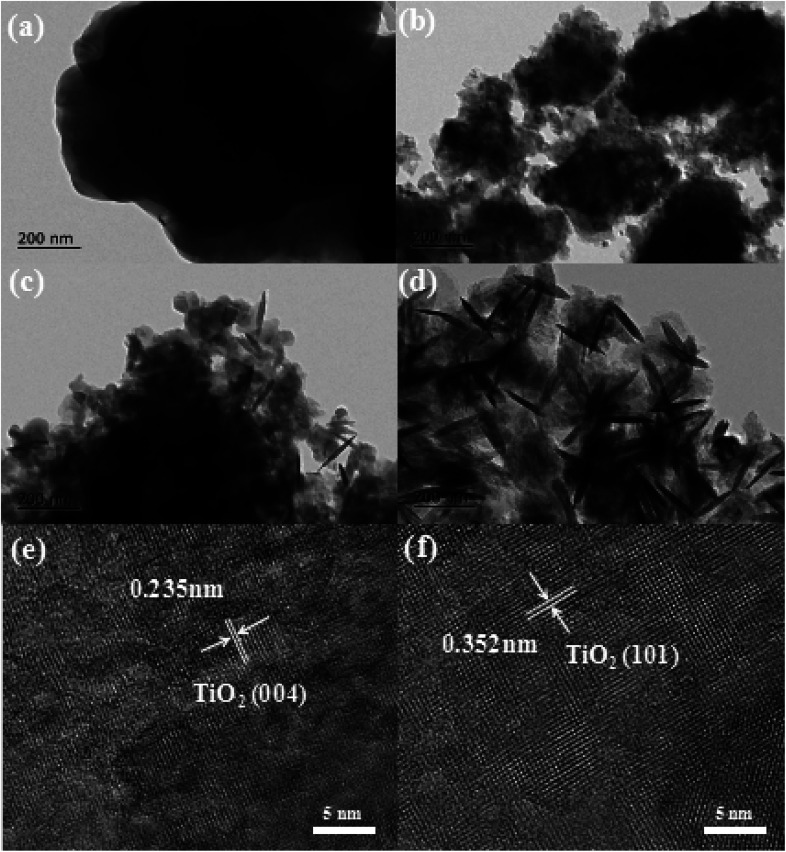
TEM images of as-prepared samples (a) T-0.5h, (b) T-1h, (c) T-3h, (d) T-4h, and HRTEM images of (e and f) T-4h.

**Fig. 4:**
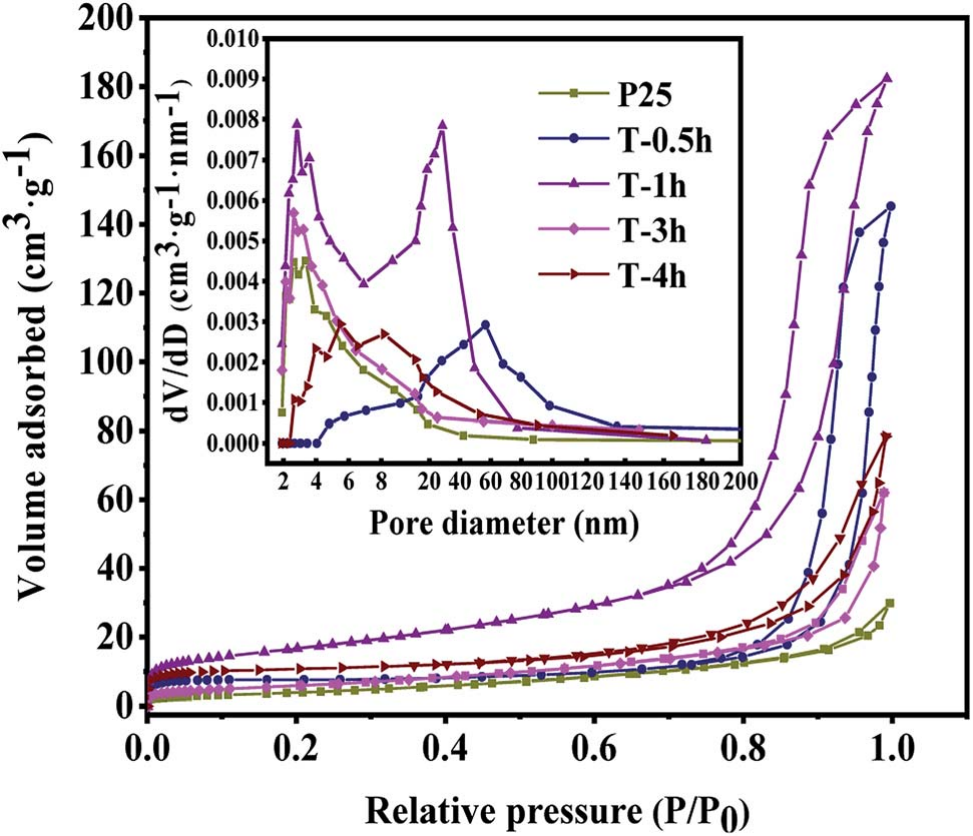
Nitrogen adsorption and desorption isotherms of T-0.5h, T-1h, T-3h, T-4h and P25. The inset is the corresponding pore size distribution.

More morphological details on prepared samples were further obtained using transmission electron microscopy (TEM) images. It can be seen from Fig. 3a that the TiOF_2_ nanoparticles are tightly packed together, which is consistent with the results observed by SEM. With the hydrothermal reaction, a small part of TiOF_2_ was transformed into TiO_2_, showing a loose stacking structure (Fig. 3b). With further reaction, it can be seen that TiOF_2_ nanoparticles are more dispersed, and more TiO_2_ nanosheets with the size of 100–200 nm are closely linked with TiOF_2_ (Fig. 3c). In Fig. 4d, it can be seen that the TiOF_2_ nanoparticles have been completely transformed in TiO_2_ nanosheets with {001} and {101} facets interlaced, with an average size of 200 nm. High-resolution TEM (HRTEM) image of T-4h (Fig. 3e) shows that the clear lattice fringes with a spacing of 0.352 nm correspond to the {101} facets, indicating that {101} facet is exposed on the surface of T-4h.^41^ The T-4h with a lattice spacing of 0.235 nm is indexed to the {004} planed (Fig. 3f), demonstrating the exposure of {001} facet on the surface of T-4h.^45,49^ Compared with the TiO_2_ with {001} facet exposure reported by most studies,^48,54^ the size of TiO_2_-{001/101} derived from coral-like TiOF_2_ is smaller. All the above results indicate that the coral-like TiOF_2_ can be transformed into cluster TiO_2_-{001/101}.

### 3.3. BET specific surface area and pore structure

The specific surface area and pore size distribution of T-0.5h, T-1h, T-3h, T-4h were analyzed by nitrogen adsorption–desorption technology. As shown in Fig. 4, according to the classification of IUPAC, T-0.5h and T-1h show a typical IV type adsorption–desorption isotherm with H1 type hysteresis ring, which represents that the samples have a mesoporous structure with close-packed spherical particles and matches the image presented by SEM. T-3h, T-4h and P25 show a typical IV type adsorption–desorption isotherm with H3 type hysteresis ring, which represents that the samples have a mesoporous structure with cracks.^22^Table 1 shows the specific surface area, pore volume and average pore size of all samples.

**Table 1:** Specific surface area, volume, mean pore size for the tested catalysts

Samples	Surface area (m^2^ g^−1^)	Pore volume (cm^3^ g^−1^)	Average pore size (nm)
T-0.5h	33.44	0.22477	37.39
T-1h	92.40	0.28203	18.88
T-3h	31.75	0.09572	17.78
T-4h	50.06	0.12127	13.82
P25	21.98	0.04630	12.67

The specific surface areas of T-0.5h, T-1h, T-3h, T-4h are 33.44, 92.4, 31.75, 50.06 m^2^ g^−1^, respectively. The specific surface area of these samples is larger than that of P25 (21.98 m^2^ g^−1^). According to the corresponding BJH pore size distribution diagram (Fig. 4 inset), the average pore size of T-4h is 13.82 nm, which is well-matched with the size of TC (1.41 nm in length, 0.46 nm in width and 0.82 nm in height).

This is consistent with the strong removal rate of the dark reaction stage in the subsequent photocatalytic degradation experiment. Besides, these uniform and small pore size mesopores are conducive to the absorption of light and multiple reflections inside the material and provide an effective transmission path for photogenerated carriers.^3^

### 3.4. FT-IR and EPR analysis

**Fig. 5:**
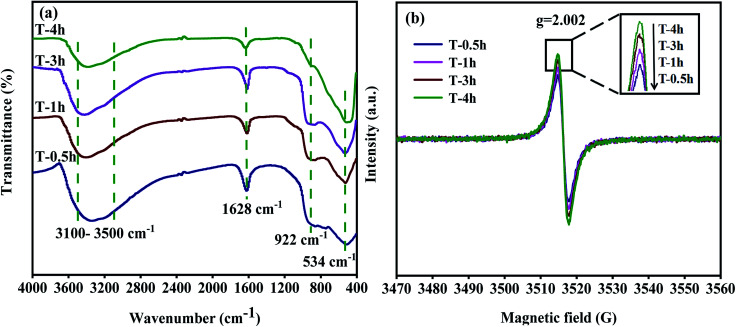
FT-IR (a) and EPR (b) spectra of different samples.

Fig. 5a shows the FT-IR spectra of photocatalysts prepared at different hydrothermal reaction times. The stretching and bending vibrations due to absorption by H_2_O and the Ti–OH group on the sample surface are found at 3100–3500 cm^−1^ and 1628 cm^−1^.^25,47^ T-0.5h, T-1h, T-3h have strong peaks at 922 cm^−1^, which is attributed to Ti–F vibration in TiOF_2_. T-4h has an only a slight peak at 922 cm^−1^, which can be attributed to the Ti–F vibration formed by adsorbed F and TiO_2_.^50^ The peak at 534 cm^−1^ is attributed to Ti–O vibration or Ti–O–Ti vibration.^52^ It should be noted that the peak of T-4h shifts negatively from 534 cm^−1^, which may be attributed to the formation of Ti^3+^ and O_v_. The increased number of O_v_ in the lattice structure changes the number of Ti atom surrounding the O atom, and the electron cloud density around a Ti atom decreased.^59^ This causes the stretching vibration absorption peak of a Ti–O bond to shift. Additional information on oxygen vacancy (O_v_) was provided by EPR spectra collected at room temperature. As displayed in Fig. 5b, the signal at *g* = 2.002 corresponds to O_v_.^63^ It can be seen that all samples show the signal of O_v_, and with the transformation of TiOF_2_ to TiO_2_-{001/101}, the signal of O_v_ in T-4h is the strongest, which indicates that there is a lot of O_v_ in T-4h, which will effectively inhibit the recombination of electrons and holes, and improve the photocatalytic activity.

### 3.5. XPS analysis

**Fig. 6:**
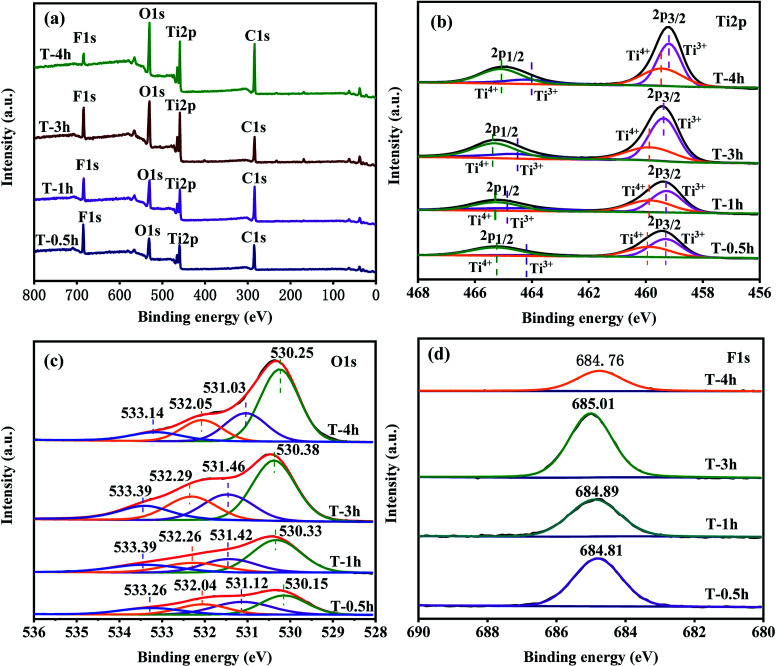
(a) Survey XPS spectra of the samples; (b, c and d) high-resolution XPS data of Ti2p, O1s and F1s for samples respectively.

The surface compositions and chemical states of pure T-0.5h, T-1h, T-3h and T-4h are displayed in Fig. 6. The survey spectra reveal signals of Ti, O, F, and C on all samples that match their respective signals from the individual spectra of TiOF_2_ and TiO_2_ (Fig. 6a), which is in accordance with the element mapping results. The existence of the peaks of element C at 284.8 eV can ascribe to surface adventitious reference carbon, which is unavoidable during the XPS measurement.^25^

From the high-resolution XPS spectrum of Ti2p in the samples (Fig. 6b). The binding energies of both Ti2p_3/2_ (from 459.48 to 459.18 eV) and Ti2p_1/2_ (from 465.28 to 464.88 eV) decreased slightly from TiOF_2_ nanoparticles to cluster TiO_2_-{001/101}. This could be attributed to the decreased bond strength of the Ti–O bond in TiO_2_ compared with the Ti–F bond in TiOF_2_ nanoparticles.^58^ Through Gauss fitting, these peaks centered at 459.31 and 464.21 eV were ascribed to Ti^3+^ species, while peaks at 459.88 and 465.26 eV were ascribed to Ti^4+^ which indicate that the Ti^3+^ has formed in the reduction process.^25^ The relative content of Ti^3+^ increases gradually with the increase of hydrothermal reaction time. The asymmetric O1s spectra were fitted to four different types of O, as shown in Fig. 6c. After Gauss fitting of all samples, the peak of the binding energy of each sample from small to large corresponds to lattice oxygen, hydroxyl oxygen, O_v_ and oxygen in adsorbed water, which can be considered as convincing evidence for the existence of O_v_.^58,63,64^

Fig. 6d shows the core-level F1s XPS spectra. The binding energy of F1s electrons shifted from 684.81 eV for T-0.5h to 684.76 eV for T-4h. The slight decrease in the F1s binding energy indicated the shifting of the Ti–F bond in the bulk TiOF_2_ nanoparticles to the surface Ti–F bonds on the TiO_2_ nanosheets.^2^

### 3.6. The optical absorption and PL analysis

**Fig. 7:**
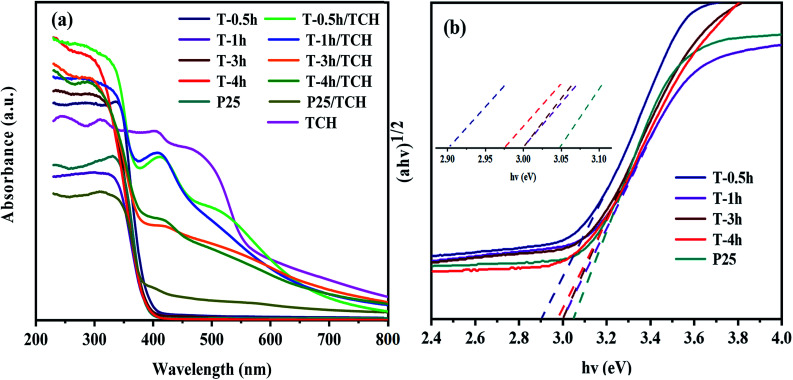
(a) UV-Vis absorbance spectra of the samples and (b) the corresponding plots of (*αhv*)^1/2^*versus hv*.

Fig. 7a shows the light absorption performance of different photocatalysts and complexes after the adsorption of TCH. All samples show different absorption in the UV region without adsorption of TCH. The amount of light absorbed in the UV region increased in the order T-1h < P25 < T-0.5h < T-3h < T-4h. However, in the visible region, only T-0.5h shows visible light response, which is attributed to the narrow band gap energy of TiOF_2_ (2.91 eV), as shown in Fig. 7b. Many researchers^22,34^ have studied the sensitization between TiO_2_ and dyes, but only a few researchers focus on the sensitization between TiO_2_ and antibiotics. Hasan *et al.*^65^ believed that the LMCT effect formed between sulfonamides and TiO_2_, because sulfonamides did not absorb visible light. Interestingly, TCH has strong absorption in the visible and UV regions as shown in Fig. 7b, which is attributed to TCH molecule possesses a delocalized π bond connected with –OH group, leading to a small energy gap between highest occupied molecular orbital (HOMO) and lowest unoccupied molecular orbital (LUMO) and thus availability for visible light absorption.^66^ When the photocatalyst adsorbs TCH, it shows different degrees of absorption in the visible light region. Wu *et al.*^66^ believed that the electron excited by visible light from HOMO to LUMO of TCH and further transferred to the conduction band of TiO_2_ was a kind of sensitization. P25/TCH has only weak light absorption in the UV and visible light region. The amount of light absorbed in the visible light region increased in the order P25/TCH < T-4h/TCH < T-3h/TCH < T-1h/TCH < T-0.5h/TCH. The novel TiOF_2_ seems to be more easily sensitized with TCH, showing the strongest absorption in the visible region, which may be attributed to the formation of intermolecular hydroxyl groups, which makes the π orbitals of TCH more easily form electronic coupling with the 3d orbitals of Ti^4+^ and Ti^3+^, resulting in the formation of tight surface complexes between TCH and TiOF_2_. Although the T-0.5h/TCH has a strong response to visible light, T-0.5h does not show the best photocatalytic activity in the degradation test of TCH, which shows that the response-ability of photocatalyst to visible light is not the only factor to evaluate the photocatalytic activity. The band gap energy (*E*_g_) of T-0.5h, T-1h, T-3h, T-4h and P25 was calculated according to the Tauc curve. The results are shown in Fig. 7b. The band gap energies of T-0.5h, T-1h, T-3h, T-4h and P25 are 2.91 eV, 3.00 eV, 3.00 eV, 2.97 eV and 3.05 eV.

PL measurements are effective to examine the separation efficiency and recombination processes of photo-generated carriers because increased photo-generated electron–hole pair recombination results in stronger luminescence intensity. Three peaks were observed in the spectra.

**Fig. 8:**
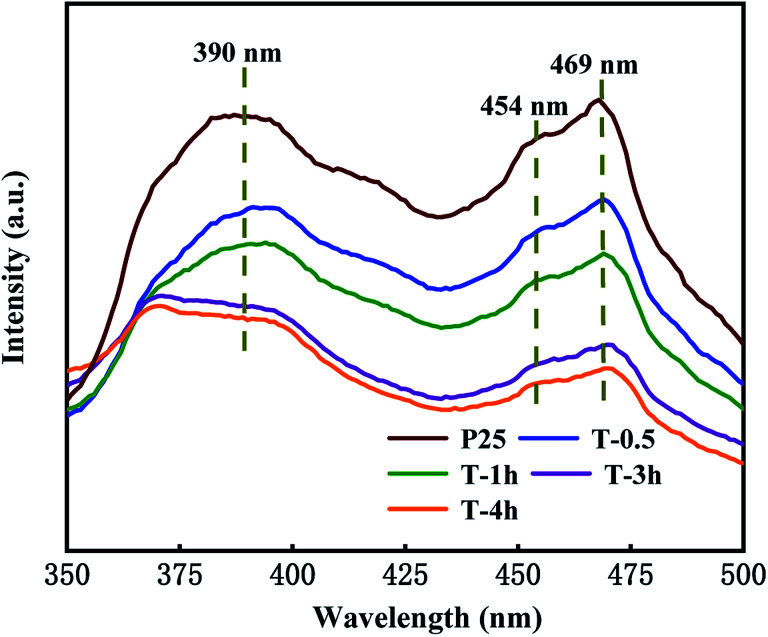
The photoluminescence spectra with the excitation wavelength *λ*_ex_ = 300 nm for T-0.5h, T-1h, T-3h, T-4h and P25.

Fig. 8 representatively shows the PL spectra of the T-0.5h, T-1h, T-3h, T-4h and P25 samples. Three peaks were observed in the spectra. The broad emission bands centered at 390 nm were ascribed to the interband transition of TiO_2_.^55^ The two peaks at 454 and 469 nm are attributed to the O_v_ and two trapped electrons. The peak of O_v_ also appears in P25, which is attributed to the mixture of rutile and anatase phases, which increases the defect density in TiO_2_ lattice.^25^ It is worth noting that the luminescence intensity of T-3h is significantly lower than that of T-0.5h and T-1h, which can be attributed to the formation of suitable heterojunction between residual TiOF_2_ and TiO_2_, which greatly reduces the recombination rate of electrons and holes. T-4h shows the lowest luminescence intensity, much lower than that of P25, this may be due to the surface heterojunction formed on {001} and {101} facets and the highest O_v_ concentration.^56,59^

### 3.7. Photocatalytic activity

**Fig. 9:**
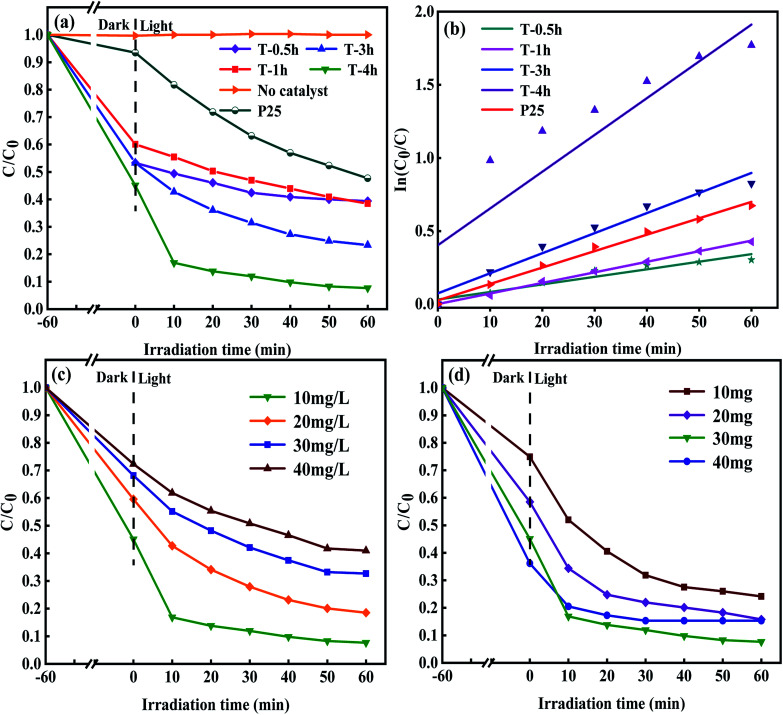
(a) Photodegradation of TCH solution (10 mg L^−1^, 100 mL) using different samples under simulated sunlight; (b) kinetic linear simulation curves of TCH solution photodegradation; (c) the effect of different concentration of TCH solution on the photocatalytic performance of T-4h (30 mg) under simulated sunlight; (d) the effect of different dosage of T-4h photocatalyst on degradation of TCH solution (10 mg L^−1^, 100 mL) under simulated sunlight.

To study the photocatalytic performance of samples, TCH solution was used as a typical antibiotic pollutant in water to simulate the solar photocatalytic degradation test. The results are shown in Fig. 9a. The photocatalysis system reacts in the dark for 60 minutes to reach the adsorption–desorption equilibrium before the sunlight. TCH has little self-degradation under sunlight. It can be seen that the samples of T-0.5h, T-1h, T-3h and T-4h have good adsorption properties. When the adsorption–desorption equilibrium was reached, the removal rates of TCH were 40%, 47%, 47% and 55%, respectively. T-4h sample has the strongest adsorption capacity for TCH. Large specific surface area, suitable pore structure and high exposure {001} facets make TCH easy to adsorb on the surface of T-4h, which lays a foundation for the subsequent photocatalytic degradation. Although the adsorption capacity of T-0.5h for TCH is stronger than that of T-1h, the total removal rate (60%) is not higher than that of T-1h (62%). This shows that the adsorption performance of photocatalyst for TCH can not dominate its ability to degrade TCH. Surprisingly, the total degradation rate of TCH by T-4h reached 83.2% only after 10 min irradiation under simulated sunlight, and 92.3% after 60 min irradiation, which was much higher than that of P25 (52%).

The degradation kinetic model was studied and the photocatalytic properties of the samples were further analyzed. Fig. 9b shows that the linear relationship between degradation time (*t*) and ln(*C*_0_/*C*) is almost a straight line, which proves that the degradation process of TCH conforms to pseudo-first-order reaction. The rate constants *k* of T-0.5h, T-1h, T-3h, T-4h and P25 were 0.00518, 0.00722, 0.01372, 0.02514 and 0.01123 min^−1^, respectively. The results showed that the degradation rate of TCH by T-3h photocatalyst was significantly higher than that of T-0.5h and T-1h, which may be due to the appropriate ratio of residual TiOF_2_ and TiO_2_ in T-3h sample, and the synergistic effect of the two substances promoted the photodegradation.

The maximum rate constant of T-4h was 2.24 times that of P25. Besides, the effects of different concentrations of TCH solution on the photocatalytic performance of the T-4h sample were studied (Fig. 9c). T-4h had the best degradation effect on the TCH solution of 10 mg L^−1^. With the increase of TCH solution concentration, the photocatalytic performance of T-4h gradually decreased. It may be that the photocatalyst is not to provide enough active reaction sites to deal with so many TCH molecules. Fig. 9d shows the degradation curve of TCH solution with different T-4h dosage (0.1 g L^−1^, 0.2 g L^−1^, 0.3 g L^−1^, 0.4 g L^−1^). When the amount of catalyst is 0.3 g L^−1^, the degradation effect of TCH is the best.

**Fig. 10:**
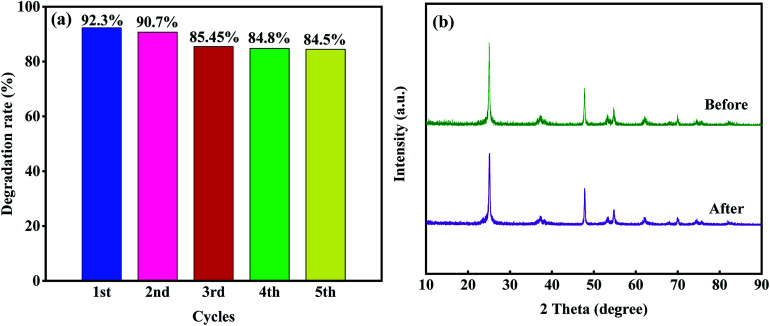
(a) Photo stability tests over T-4h for TCH degradation; (b) XRD patterns of T-4h before and after five cycling runs; (c) effect of different scavengers on the degradation of TCH (10 mg L^−1^, 100 mL) efficiencies over T-4h.

However, when the dosage of T-4h reached 0.4 g L^−1^, the photocatalytic effect decreased which may be due to the aggregation of excessive catalysts, which makes the solution turbid and leads to photon scattering, thus reducing the photocatalytic rate.^16^ The stability and reusability of photocatalyst are important factors affecting its practical application. Five consecutive cyclic photodegradation tests were carried out on sample T-4h, and the results are shown in Fig. 10a. After five cycles, the degradation rate of sample T-4h in simulated sunlight decreased from 92.3% to 84.5%, indicating that sample T-4h has a high reuse rate and photocatalytic performance.

As shown in Fig. 10b, after five cycles, the peak intensity of T-4h photocatalyst decreased, but the position of diffraction peak was the same as that before reaction, indicating that T-4h has high chemical stability.

**Fig. 11:**
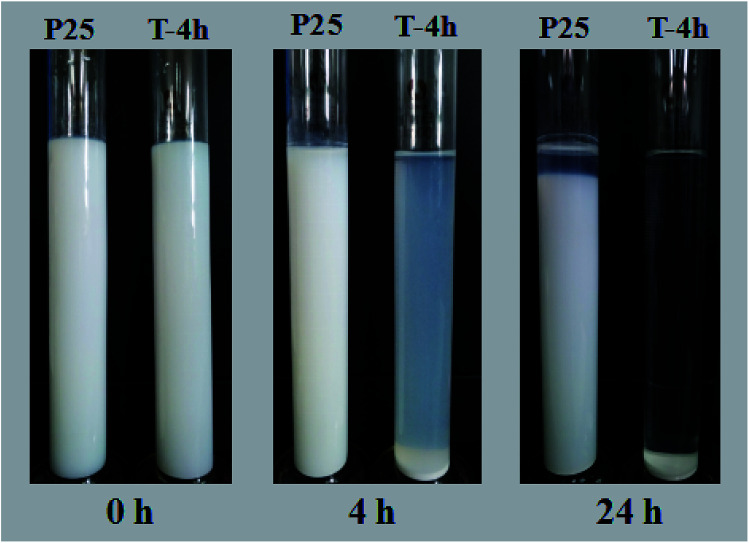
Photographs of P25 suspension and T-4h suspension in deionized water at pH 7.0 at different settling times.

Besides, the settlement performance of T-4h and P25 were further studied. The digital images of P25 and T-4h suspensions in deionized water at pH 7.0 were taken at different sedimentation times, as shown in Fig. 11. P25 nanoparticles were well dispersed in water and settled very slowly. The supernatant was still very turbid after 24 h of sedimentation. The settling behavior of T-4h was distinctly different from that of P25. Most of the composites was settled in 4 h leaving a small fraction of T-4h suspended in the supernatant, which became clear after 24 h of sedimentation. This shows that T-4h is more conducive to the practical application of wastewater treatment than P25.

### 3.8. Photocatalysis mechanism

In general, hydroxyl radicals (·OH), superoxide radicals (·O_2_^−^) and holes (h^+^) can be found in the photocatalytic degradation process. For this reason, many scavengers, such as benzoquinone (BQ, ·O_2_^−^), *tert*-butyl alcohol (*t*-BuOH, ·OH), methanol (MT, h^+^) and so on, were introduced to carry out free radical trapping experiments.

**Fig. 12:**
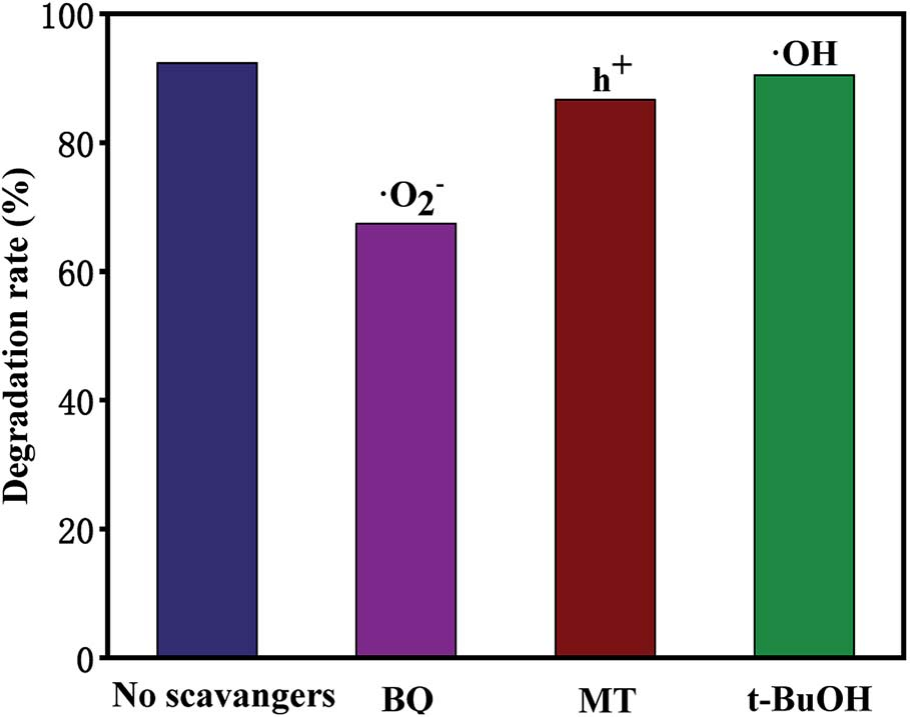
Effect of different scavengers on the degradation of TCH (10 mg L^−1^, 100 mL) efficiencies over T-4h.

**Fig. 13:**
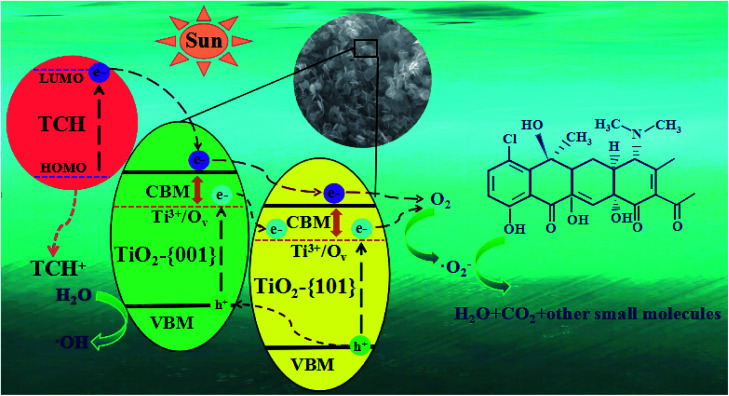
Schematic illustration of the photocatalytic mechanism for TCH removal over the T-4h photocatalyst.

It can be seen from Fig. 12 that under simulated sunlight irradiation, the introduction of BQ drastically decreased photodegradation efficiency of TCH from 92.3 to 67.4%, suggesting that ·O_2_^−^ played a key role in the photodegradation. When MT was added, the total degradation rate decreased by 5.65%, indicating that h^+^ was the secondary substance affecting the photocatalytic degradation. After adding *t*-BuOH, the photocatalytic degradation was almost not inhibited, and the total removal rate decreased by 2%, indicating that ·OH did not play a major role in the degradation. Based on the experimental results, the possible mechanism of outstanding photocatalytic activity of T-4h was proposed (Fig. 13). Firstly, the {001} facets of TiO_2_-{001/101} has a strong adsorption capacity for TCH.

Furthermore, the π orbital of TCH may form electronic coupling with the 3d orbital of Ti^4+^, resulting in a surface complex between TCH and TiO_2_-{001/101}.^67^

TCH was degraded simultaneously in two pathways. In the first path, electrons are excited from the HOMO level of TCH to LUMO level under simulated sunlight, and then rapidly injected into the conduction band maximum (CBM) position of TiO_2_-{001} facets.^39^ However, TCH lost its electrons and remained a stable product (TCH^+^). Because the CBM of TiO_2_-{001} facets is more negative than that of TiO_2_-{101} facets. The electrons on CBM of TiO_2_-{001} facets can migrate to CBM of TiO_2_-{101} facets, and eventually react with O_2_ adsorbed on the TiO_2_-{101} facet to form ·O_2_^−^ to oxidize TCH and TCH^+^.

In the second path, the doping of Ti^3+^/O_v_ can form new states at the bottom of the TiO_2_-{001/101} CBM. Under simulated sunlight irradiation, the {001} and {101} facets of TiO_2_ produce electron–hole pairs respectively. The CBM and Ti^3+^/O_v_ state potentials of TiO_2_-{001} facets are negative than that of TiO_2_-{101} facets, respectively, allowing the holes of TiO_2_-{101} CBM partly transfer to that of TiO_2_-{001} CBM, and the electrons in Ti^3+^/O_v_ states of TiO_2_-{001} facets transfer to the Ti^3+^/O_v_ states of TiO_2_-{101} facets.^67^ This process resulted in efficient space separation of photo-induced charge carriers to suppress recombination. Similar to the first pathway, electrons in the Ti^3+^/O_v_ states can interact with O_2_ to form ·O_2_^−^ which can oxidize TCH and TCH^+^. The remaining holes can react with H_2_O to form ·OH and oxidize TCH with ·O_2_^−^.

## 4. Conclusions

In summary, coral-like TiOF_2_ can be synthesized by a one-step hydrothermal method, and with the extension of hydrothermal reaction time, cluster TiO_2_-{001/101} with the smaller size can be obtained. The prepared cluster TiO_2_-{001/101} exhibits excellent TCH degradation activity under simulated sunlight. The sensitization between TCH and cluster TiO_2_-{001/101} can induce strong visible light absorption. It is worth noting that the photocatalyst has good stability, repeatability, and sedimentation, which is very important in practical application. This work opens up a new way to explore the TiOF_2_ and provides a new idea for the degradation of TCH.

## Conflicts of interest

There is no conflict of interests exiting in the manuscript submission, and it is approved by all of the authors for publication. All the authors listed have approved the manuscript to be enclosed.

## Supplementary Material
